# Hydrogen peroxide room disinfection – ready for prime time?

**DOI:** 10.1186/s13054-015-0915-8

**Published:** 2015-05-08

**Authors:** Benedikt D Huttner, Stephan Harbarth

**Affiliations:** Infection Control Program and Division of Infectious Diseases, Geneva University Hospitals and Faculty of Medicine, Rue Gabrielle-Perret-Gentil 4, 1205 Geneva, Switzerland

## Abstract

Non-manual techniques for terminal disinfection of hospital rooms have gained increasing interest in recent years as means to reduce transmission of multidrug-resistant organisms (MDROs). A prospective crossover study by Blazejewski and colleagues in five ICUs of a French academic hospital with a high prevalence of MDRO carriers showed that two different hydrogen peroxide (H_2_O_2_)-based non-touch disinfection techniques reduced environmental contamination with MDROs after routine cleaning. This study provides further evidence of the ‘in use’ bioburden reduction offered by these techniques. Before H_2_O_2_-based non-touch disinfection can be recommended for routine clinical use outside specific outbreak situations, further studies need to show whether the environmental contamination reduction provided by these techniques is clinically relevant and results in reduced cross-infections with MDROs.

## Commentary

Many people associate the smell of hospitals with the smell of disinfectants, and few people would feel comfortable being hospitalized in a facility that is not free of visible dirt. Since the close environment of the patient gets contaminated with microorganisms originating from its human occupant, hospital rooms usually undergo terminal cleaning with conventional ‘hands-on’ disinfection techniques (by mechanically applying a disinfecting product) after the patient has left the room. These techniques are highly operator-dependent and difficult to standardize and leave room for error. In recent years, non-manual disinfection techniques (sometimes called ‘non-touch disinfection’) via hydrogen peroxide (H_2_O_2_) vapor or aerosol, ultraviolet light, or antimicrobial surfaces have therefore gained interest.

In a recent article in *Critical Care*, Blazejewski and colleagues [1] describe the results of a prospective crossover study in a French tertiary care center examining the impact of terminal disinfection with two different non-manual H_2_O_2_ disinfection techniques on environmental contamination with multidrug-resistant organisms (MDROs). The authors should be congratulated for the rigorous setup and environmental sampling in this study.

After routine terminal cleaning with a quaternary ammonium compound and a sodium hypochlorite solution, rooms were disinfected by either H_2_O_2_ vapor or aerosolized H_2_O_2_ combined with peracetic acid during 6 weeks with a switch to the other methodology for another 6 weeks. The primary outcome was environmental contamination of a room with any MDRO defined as methicillin-resistant *Staphylococcus aureus* (MRSA), *Pseudomonas aeruginosa* resistant to ceftazidime or imipenem, extended spectrum β-lactamase Enterobacteriaceae (ESBL-E), imipenem-resistant *Acinetobacter baumannii*, or vancomycin-resistant enterococci. Extensive environmental sampling of 24 high-touch surfaces was performed after patient discharge, after routine terminal cleaning, and after H_2_O_2_ disinfection. Both H_2_O_2_ techniques reduced environmental MDRO contamination from 11 of 182 rooms after terminal cleaning to 1 of 182 rooms after H_2_O_2_ disinfection.

How should those findings be interpreted, and do the results support the widespread use of non-manual H_2_O_2_-based disinfection techniques? First of all, it is worth mentioning that the setting in which the study was carried out is somewhat atypical. In the study hospital, all 46 rooms in the five included ICUs were single-occupancy rooms, hardly the standard situation in most hospitals. Apart from providing excellent conditions for infection control, this setup greatly facilitates the use of H_2_O_2_ techniques that can be applied only in empty rooms because of toxicity risk. Thus, ICUs with a less advantageous architectural setup may find it hard to introduce H_2_O_2_ terminal disinfection into routine practice.

A further particularity worth pointing out is the high prevalence of MDRO carriage among room occupants. A strikingly high proportion (42%) of all patients in this study was colonized with ESBL-E (24%), resistant *P. aeruginosa* (10%), MRSA (8%), or imipenem-resistant *A. baumanii* (6%). This high prevalence could be related to silent MDRO transmission clusters or a very active MDRO screening policy. Despite this high prevalence of MDRO carriers and extensive environmental screening of surfaces, only 23 (1.5%) of 1,456 sampled surfaces and 15 (8%) of 182 rooms were MDRO-positive after patient discharge, before any disinfection. Interestingly, the environmental MDRO contamination reduction provided by H_2_O_2_ disinfection after routine cleaning was uniquely attributable to a reduction in ESBL-E (indeed, none of the other MDROs was identified after routine cleaning), which were isolated mostly from sinks, where other environmental infection control measures may be easily applicable [2]. With regard to ESBL-E, there are probably differences between species in the degree of environmental contamination (higher for *Klebsiella* spp. than for *Escherichia coli*), but unfortunately the ESBL-E species were not reported in this study [3].

Surprisingly, only 27 of 74 MDRO carriers were identified as such at ICU admission, suggesting that a non-negligible number of patients may have acquired MDROs during their ICU stay despite the enhanced terminal disinfection. This confirms that other routes of transmission (for example, through the hands of health-care workers) play an important role [4].

Given the results of the present and numerous other studies, there is little doubt that MDROs contaminate the environment and that H_2_O_2_-based techniques can reduce this environmental contamination [5-7]. H_2_O_2_-based techniques thus improve ‘cleanliness’, but does it matter? Environmental contamination is only a surrogate marker and reducing it cannot be a goal *per se* given the significant resources in time and money associated with these techniques. The real question is whether the environmental contamination reduction offered by non-manual disinfection techniques compared with conventional disinfection results in significantly reduced MDRO cross-infection and this question remains unanswered. We are still low on the ‘evidentiary hierarchy’ outlined by McDonald and Arduino [8]. Most studies aiming for the higher level of the ‘evidentiary hierarchy’ pyramid have significant methodological limitations and more research in this area is needed [8-11].

## Conclusions

The study by Blazejewski and colleagues provides further evidence that non-manual disinfection techniques based on H_2_O_2_ can reduce environmental contamination with MDROs compared with standard cleaning alone. However, solid evidence that these techniques ultimately reduce MDRO cross-infection is lacking. Given the considerable cost associated with these techniques and the fact that the majority of transmission events in non-epidemic settings are probably not mediated by the environment, it seems advisable for now to continue to invest most effort into traditional infection control measures such as hand hygiene and wait for further evidence before introducing these techniques into routine care.

## Abbreviations

ESBL-E: Extended spectrum β-lactamase Enterobacteriaceae
H_2_O_2_: Hydrogen peroxide
MDRO: Multidrug-resistant organism
MRSA: Methicillin-resistant *Staphylococcus aureus*
